# Descriptive Epidemiology and Phylodynamics of the “First Wave” of an Outbreak of Highly Pathogenic Avian Influenza (H5N1 Clade 2.3.4.4b) in British Columbia and the Yukon, Canada, April to September 2022

**DOI:** 10.1155/2024/2327939

**Published:** 2024-02-29

**Authors:** Cassandra L. Andrew, Shannon L. Russell, Michelle Coombe, James E. A. Zlosnik, Kevin S. Kuchinski, Jessica Caleta, Chris Fjell, Yohannes Berhane, Victoria Bowes, Tony Redford, Caeley Thacker, Laurie Wilson, Maud Henaff, N. Jane Harms, Agatha Jassem, Jolene Giacinti, Catherine Soos, Natalie Prystajecky, Chelsea Himsworth

**Affiliations:** ^1^Department of Medicine, School of Population and Public Health, University of British Columbia, 2206 East Mall Vancouver, B.C. V6T 1Z3, Vancouver, Canada; ^2^British Columbia Centre for Disease Control, 655 W 12th Avenue Vancouver, B.C., V5Z 4R4, Vancouver, Canada; ^3^Department of Pathology and Laboratory Medicine, University of British Columbia, 2211 Wesbrook Mall, Vancouver, B.C. V6T 1Z7, Canada; ^4^Animal Health Centre, British Columbia Ministry of Agriculture and Food, 1767 Angus Campbell Road, Abbotsford, B.C. V3G 2M3, Canada; ^5^Public Health Agency of Canada (PHAC), National Microbiology Laboratory (NML), 1015 Arlington St., Winnipeg, Manitoba, R3E 3P6, Canada; ^6^Canadian Food Inspection Agency, National Centre for Foreign Animal Disease, 1015 Arlington Street, Winnipeg MB R3E 3M4, Canada; ^7^British Columbia Ministry of Forests, P. O. Box 9338 Stn Prov Govt Victoria, Victoria, B.C., V8W 9M3, Canada; ^8^Canadian Wildlife Service, Environment and Climate Change Canada, Pacific Wildlife Research Centre, 5421 Robertson Road, Delta, B.C. V4K 3N2, Canada; ^9^Government of Yukon, Department of Environment, Animal Health Unit, 10 Burns Road, Whitehorse YT Y1A 4Y9, Canada; ^10^Ecotoxicology and Wildlife Health Division, Science and Technology Branch, Environment and Climate Change Canada, 115 Perimeter Road, Saskatoon, SK S7N 0X4, Canada; ^11^Canadian Wildlife Health Cooperative British Columbia, 1767 Angus Campbell Road, Abbotsford, B.C. V3G 2M3, Canada

## Abstract

Highly pathogenic avian influenza (HPAI) is a viral disease that causes significant rates of morbidity and mortality in domestic poultry and wild birds, with occasional spillover into mammals, including humans. Beginning in November 2021, Canada experienced its longest and largest outbreak of HPAI in history. A portion of this outbreak (H5N1, clade 2.3.4.4b) occurred in western Canada, specifically in British Columbia (B.C.) and the Yukon, between April 12 and September 11, 2022, which was classified as the “first wave” in this region. Wild birds and mammals identified through passive surveillance and suspect domestic poultry flocks were screened for avian influenza virus (AIV), typed H5 by qPCR, and positive cases were whole genome sequenced. Descriptive epidemiological and phylodynamic analyses were performed to: (1) understand the taxonomic and geographic extent of wild species involved; and (2) examine the origins and probable transmission networks of HPAI viruses introduced into B.C./Yukon by comparing local viruses with those circulating elsewhere in North America. This outbreak included 21 species of wild birds, 2 species of wild mammals, 4 commercial, and 12 domestic small flock infected premises. Canada geese (*Branta canadensis*) and bald eagles (*Haliaeetus leucocephalus*) were the most common wild species detected. We demonstrate that north-south avian migration via the Pacific Flyway is the probable route of multiple incursions into this region. Phylogenetic analysis of the hemagglutinin (HA) segment revealed that the B.C./Yukon viruses detected formed five distinct genetic clusters which were maintained across the whole genome. Although, the genome segments were predominantly Eurasian in origin, NP and PB2 segments from all samples, as well as NS and PB1 segments from Cluster 3, had North American origins. Overall, we demonstrate the utility of genomic epidemiology to inform HPAI transmission dynamics across Western Canada and discuss potential knowledge gaps that exist in passive surveillance strategies for HPAI.

## 1. Introduction

Highly pathogenic avian influenza (HPAI) is a viral disease that causes significant morbidity and mortality in domestic poultry and wild birds, with occasional spillover into mammals, including humans. The emergence of the highly pathogenic A/goose/Guangdong/1/1996 (Gs/GD) H5N1 influenza A lineage in domestic poultry, followed by its reassortments with low-pathogenicity subtypes, has contributed to the global dissemination of various avian influenza viruses (AIVs) affecting both domestic and wild bird species [1], and recurrent, ongoing threats to human health [2]. Outbreaks in poultry have significant consequences for economies [3] and food security globally, and in North America specifically [4]. Meanwhile, HPAI outbreaks in wildlife may impact avian conservation—for example, Nemeth et al. [5] found that HPAI infection in bald eagles (*Haliaeetus leucocephalus*) in two Southeastern American states, Georgia and Florida, resulted in high rates of mortality as well as decreased hatchling/nest success. Furthermore, multiple viral mutations have implications for the emergence of an influenza virus with human pandemic potential [6].

Wild Anseriformes (ducks and geese) and Charadriiformes (gulls, terns, and other shorebirds) are the main reservoirs for influenza A viruses [7], including HPAI H5N1, and are responsible for translocating AIVs between geographic locations during local movement and transcontinental migration [8], and particularly along a north-south axis within North America [9]. For example, the Pacific Flyway spans from Alaska (USA) to Patagonia (Argentina), through the westernmost portion of the Americas, and overlaps in certain areas with other globally distributed migratory flyways, particularly north of 60° parallel and in Central and South America [10]. Rates of detection and mortality events associated with H5N1 clade 2.3.4.4b have been increasing in global breadth and intensity since 2021 [11], and wild reservoir birds are the main source of infection for domestic poultry [12–15]. For this reason, surveillance in wildlife and poultry are equally important for both risk assessment and outbreak detection, as well as for understanding the epidemiology of outbreaks once they occur.

HPAI has previously been detected in B.C. in 2004 (H7N3) [16] and 2014 (H5N2 with H5N8 segments (polymerase basic 2 (PB2), polymerase acidic (PA), hemagglutinin (HA), matrix (M), and nonstructural (NS); [17]; H5N1 [18]), primarily in domestic poultry and a few opportunistic samples from wildlife. HPAI has not previously been detected in the Yukon. The most recent outbreak of HPAI H5N1 in North America was first detected in wild birds (black-backed gulls (*Larus marinus*)) and assorted domestic poultry and captive wild birds in St. John's Newfoundland and Labrador, Canada, in November 2021 [19]. Detections were subsequently made in migratory and resident wild birds, as well as domestic poultry facilities in the southeastern United States, and swept across Eastern and Central Canada throughout 2022 [20–22]. While there was a single detection of HPAI H5N1 2.3.4.4b in a bald eagle in B.C. in February 2022, this may have been a genetically isolated event [23] because it was phylogenetically distinct from the outbreak cases described herein.

Following this first isolated incursion, there were two “waves” of HPAI infections throughout B.C. and the Yukon in 2022–2023, the first occurring from April 2022 to early September (management cutoff was set to September 11, 2022), and then the second from the latter half of September 2022 until present (June 2023). In this study we focus on the first wave, and therefore the April 12, 2022 case from a commercial poultry flock in Enderby was the first case we identified in B.C. The first cases detected in the Yukon were a trumpeter swan (*Cygnus buccinator*) and a Canada goose (*Branta canadensis*) identified on May 2, 2022.

Understanding the molecular characteristics of HPAI viruses can further resolve the dynamics of an outbreak, revealing patterns of transmission, diversity of circulating viruses and novel reassortants. Examining the relatedness of the B.C./Yukon viruses to the first introduction of HPAI H5N1 detected in Newfoundland [19] provides key insights into the origin of the HPAI viruses introduced in Western Canada several months later. Characterizing the extent of genetic diversity in an outbreak of HPAI has important implications for risk assessment monitoring and the association of specific viral genotypes with more severe clinical disease in wild and/or domestic birds, mammals, and humans.

The goal of this study is to describe the taxonomic, spatiotemporal, and phylogenetic characteristics of wildlife and poultry collected through passive surveillance during the “first wave” of the 2022 HPAI outbreak in B.C. and the Yukon.

## 2. Materials and Methods

### 2.1. Passive Surveillance Programs

Samples and data from wildlife that were included in this study were obtained from the HPAI passive surveillance programs mounted by the Governments of B.C. and the Yukon. In B.C., dead wild birds can be reported via the “Interagency Wild Bird Mortality Hotline” [24] or to the Ministry of Forest, Lands, and Natural Resource Operations or to the Canadian Wildlife Service (CWS)–Environment and Climate Change Canada (ECCC) directly. Subsequently, biologists arrange transport of carcasses to the B.C. Ministry of Agriculture Animal Health Centre, Abbotsford, BC where oropharyngeal/nasopharyngeal and rectal/cloacal swabs are obtained. In the Yukon, sick or dead wild birds and wildlife were reported by the public to the Conservation Officer Services Branch or the Animal Health Unit in the Department of Environment, Yukon Government. Oropharyngeal/nasopharyngeal and rectal/cloacal swabs samples were collected from wildlife found dead or from wildlife displaying symptoms suspicious of HPAI prior to death or euthanasia. Swab samples were collected by the Animal Health Unit staff and placed into transport media and frozen at −20°C.

Domestic birds were swabbed by veterinarians/staff from the Canadian Food Inspection Agency (CFIA) in the field (on farm) upon first suspicion of an outbreak. There were no domestic bird cases detected in the Yukon.

All swabs were placed in viral transport medium and refrigerated prior to submission to the B.C. Centre for Disease Control (BCCDC). Yukon samples were shipped and submitted frozen.

### 2.2. Data Collection

For all samples, the data collected included death date, collection date, sample type (oropharynx, nasopharynx, cloacal), and a description of the approximate geographic location where the animal was found. GPS coordinates were recorded for most samples. Where they were not, ecoprovince was estimated from the location description. For wild birds and mammals, the species, ecoprovince, ecoregion, bird conservation region (BCR), sex, and age or age class (where possible on necropsy) were recorded. In some cases, only a subsample of dead birds were retrieved and sampled from larger mortality events, in which case the total estimated number of animals affected was also recorded. Found date was used for death date in the analysis when death date was not recorded or available. For domestic birds, an infected premise (IP) number was assigned by the CFIA.

### 2.3. AIV PCR, Subtyping, and Sequencing

Samples were screened for AIV using a reverse transcription real-time quantitative polymerase chain reaction (RT-qPCR) assay targeting a conserved region in the matrix (M) gene [25], then subtyped for H5 using an in-house designed BCCDC lab-developed test targeting the HA gene segment of Influenza A virus. Samples were considered negative at CT values > 40, and “indeterminate” if CT values were between 36–40. Positive samples (CT < 36) were submitted for whole genome sequencing (WGS).

WGS libraries were generated using previously described methods with modifications to primers to improve amplicon diversity and specificity as well as library preparation efficiencies [26–28]. WGS analysis was performed using the Nextflow pipeline FluViewer-nf v0.0.9, which leverages the FluViewer tool [29] to analyze AIV sequences. Consensus sequences were generated by aligning reads (bwa v0.7.17) to a curated reference database. HA and NA subtypes were determined based on the subtype associated with the best match in the reference database. *In silico* HPAI detection was also performed [30] to identify the highly basic PLREKRRKRGLF motif in the HA cleavage site of successfully sequenced outbreak specimens, providing molecular evidence of HPAI in the PCR-positive bird/animal samples. For inclusion in the phylogenetic analysis, sequences required a minimum 20x depth and 90% coverage across each segment.

### 2.4. HPAI Case Definition

The first detection of HPAI in B.C. or the Yukon that occurred during the study period was in a commercial poultry flock on April 12, 2022; the last detections identified were from two Canada geese (*B. canadensis*) that were found dead on September 7, 2022. Confirmed HPAI cases that were included in the descriptive epidemiological analysis were samples that screened positive for avian influenza on FluANat (PCR) and were subsequently confirmed H5 on PCR or WGS. Cases were excluded if they were not successfully subtyped H5 or if their death date occurred after September 11, 2022. This management cutoff date was identified to better classify the two separate waves of HPAI as it preceded the start of fall migration for most dabbling ducks in this region, and because post-outbreak surveillance (conducted by the CFIA) on domestic premises infected during the spring/summer period had finished by this time.

### 2.5. Descriptive Epidemiology

Analyses for the descriptive epidemiology were performed in R (4.2.1 GUI 1.79 High Sierra build (8095)) and RStudio (2022.07.2 Build 576) to describe the distribution of infection across space and time, as well as the taxonomic classification. ArcMap Pro (v 3.0.0 2023) was used for developing maps and analyzing geographic trends using point data, proportional pie charts, and ecoprovince classification. B.C. ecoprovince divisions were used in order to divide the geographical landscape into potentially biologically relevant divisions for avian habitat use based on consistent climatic processes, oceanography, relief and regional landforms [31]. The Yukon was evaluated as a single geographical division. In order to better understand what types of birds were predominantly impacted, we grouped birds into six functional groups largely based on management strategies [32, 33]: “Domestic commercial,” “Domestic small flock,” “Landbirds,” “Raptors,” “Waterbirds,” and “Waterfowl”. We also included a category for “Mammals”. Due to low numbers of detections, one seabird was grouped with “Waterbirds” and five “Corvids” were grouped with “Landbirds”. Commercial poultry flocks are those which sell poultry products, and small flock (or noncommercial) poultry premises are those with less than 300 birds or which do not sell their poultry products or have limited sales locally [34]. Death month (i.e., April, May, June, July, August, or September) was used to classify the temporal extent for this portion of the analysis.

### 2.6. Phylogenetic Analysis

Phylogenetic trees (based on nucleotide sequences) for all segments were constructed using fluflo v0.1.1 (https://github.com/BCCDC-PHL/fluflo), a Nextflow wrapper for Nextstrain implementation, containing IQTREE v2.1.4_beta, with the same parameters applied to all samples. Phylogenetic trees were visualized using Nextstrain v13.0.0, Auspice v2.40.0. HA-specific phylogenetic trees contextualized with North American HPAI sequences consisted of sequences from B.C./Yukon, as well as publicly available H5Nx sequences from Canada (*n* = 9) and the U.S.A. (*n* = 767) downloaded from the Global Initiative on Sharing All Influenza Data (https://www.gisaid.org/), or GISAID, database [35] with specimen collection dates between September 1, 2021 and September 15, 2022. For whole genome analyses examining maintenance of cluster membership, phylogenetic trees were constructed for all eight gene segments individually (HA, M, neuraminidase (NA), nucleoprotein (NP), NS, PA, polymerase basic 1 (PB1), PB2) and each tree was rooted on the fully Eurasian H5N1 virus introduced in Newfoundland in December 2021 (A/chicken/NL/FAV0033/2021). Only sequences that met the appropriate coverage thresholds for inclusion were incorporated into the trees. Reassortants were identified by constructing phylogeny for each segment comprised of local (B.C./Yukon) and global GISAID H5N1 sequences collected between September 15, 2021 and September 15, 2022 and examining the genetic distance (based on SNPs) of B.C. sequences relative to the fully Eurasian NFL genotype, as well as related global sequences that fall into North American and Eurasian lineages. In all phylogenetic analyses, sequences were defined by the date the specimen was collected (“Collection date”) rather than the “Date Died” due to timing of the analyses.

## 3. Results

### 3.1. Descriptive Epidemiology

Between April 12 and September 11, 2022, we detected HPAI in 128 wild animals and 17 infected domestic poultry premises (IP). Twenty-one (21) species of wild birds and two species of mammals tested positive for H5 HPAI (Table 1). To the best of our knowledge, we report the first known detections of HPAI in wild birds in the Yukon. The wild bird species most frequently detected as positive for HPAI in this outbreak were Canada geese (*n* = 48, 32.9%), bald eagles (*n* = 25, 17.1%), and great horned owls (*Bubo virginianus*; *n* = 12, 8.2%). There were 4 commercial and 12 domestic small flock IPs detected during this wave of the outbreak.

### 3.2. Geo-Temporal Distribution

**Figure 1:**
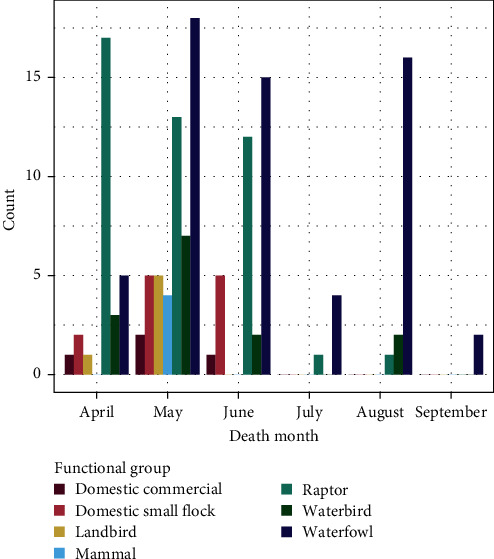
Number of wild animals (birds and mammals) or domestic infected premises (IP) that tested positive for highly pathogenic avian influenza (HPAI; H5N1 clade 2.3.4.4b) in British Columbia (B.C.) and the Yukon, Canada, during the “first wave” of the outbreak in this region, between April 12, 2022 and September 11, 2022. Data are categorized per “Functional group” (“Domestic commercial,” “Domestic small flock,” “Landbird,” “Mammal,” “Raptor,” “Waterbird,” or “Waterfowl”), per “Death Month” (April, May, June, July, August, or September).

The majority of HPAI cases occurred in May (*n* = 30, 20.8%) (Figure 1). All cases from the Yukon (all wild animals) occurred in May (*n* = 7, 4.9%), and all mammal cases (*n* = 4, 2.8%) were detected in May. We saw a higher rate of detection in “Raptors” in the first half of the outbreak (April, May, and June), and a transition to higher rates and proportions of detection in “Waterfowl” as the outbreak went on. By September, only “Waterfowl” infections were detected.

**Figure 2:**
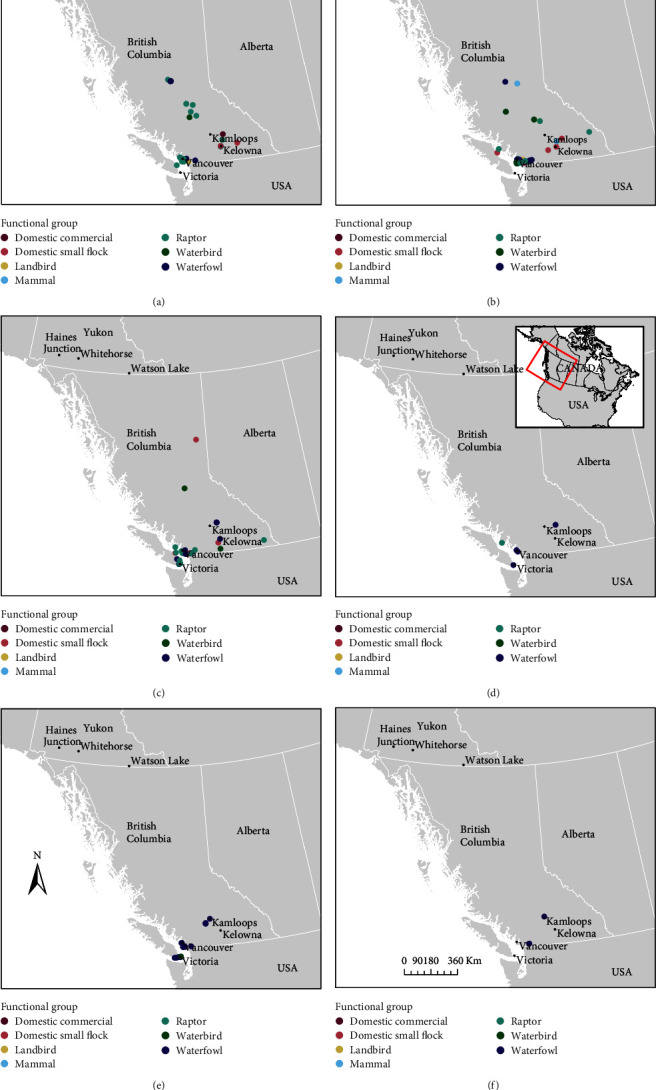
Maps demonstrating point locations where animals (birds and mammals) tested positive for highly pathogenic avian influenza (HPAI; H5N1 clade 2.3.4.4b) in British Columbia (B.C.) and the Yukon, Canada, during the “first wave” of the outbreak in this region, between April 12, 2022 and September 11, 2022, per month. Point data are categorized by “Functional Group” (“Domestic commercial,” “Domestic small flock,” “Landbird,” “Mammal,” “Raptor,” “Waterbird,” or “Waterfowl”) and by “Death month” (April (a), May (b), June (c), July (d), August (e), and September (f)).

Most cases came from the ecoprovince “Georgia Depression” (GED) (*n* = 89, 61.8%) (Figure 2(a)–2(f) and Figures S1 and S2), where the largest urban centre in B.C. (Vancouver) is located, as well as dense farmland within the Fraser Valley, the southern portion of Vancouver Island including the city of Victoria, and the Gulf Islands. While largely anthropogenically modified, the area does contain B.C.'s largest nutrient rich estuary which attracts both migrant and overwintering birds, as well as diverse coastal rainforest flora, and 90% of the province's avian diversity [26]. The second greatest number of samples came from the ecoprovince “Southern Interior” (SOI) (*n* = 24, 16.7%) which includes the cities of Kamloops and Kelowna and the dense farmland of “the Okanagan”. There was a greater geographic extent of the outbreak at the peak of the outbreak, April through June, including the Yukon, and Central and Northern B.C. (Figure 2) compared to the latter months (July–September). In general, the more cases there were in total, the more spread out they were geographically, and case detections were made in a greater diversity of functional groups (Figure 2 and Figures S1 and S2). Thus, there was a temporal effect evident in this outbreak (most cases occurred in May), but the proportions of functional groups (e.g., Waterfowl vs. Raptor) were randomly distributed at the level of ecoprovinces (Figures S1 and S2). An exception to this was observed in a group of American white pelicans (*Pelecanus erythrorhynchos*), which are “Waterbirds,” that occurred in the “Central Interior” (CEI) ecoprovince in April (*n* = 1), May (*n* = 4), and June (*n* = 1), within their summer breeding range [36].

#### 3.2.1. Hemagglutinin (HA)-Specific Phylogenetic Analysis

**Figure 3:**
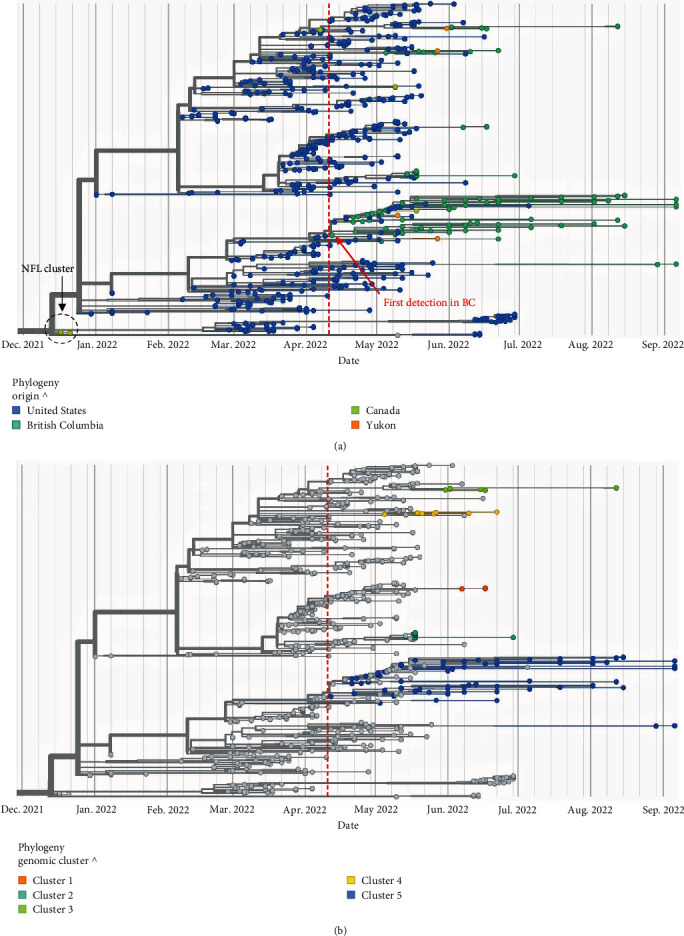
Hemagglutinin (HA)-specific phylogenetic analysis of H5N1 detections in British Columbia (B.C.) and Yukon contextualized by H5N1 sequences from other parts of Canada and the United States between September 2021 and September 2022. H5N1 sequences displayed by (a) country of origin and time (swab collection date) since the first N. American detection in Dec. 2021 (in Newfoundland) and (b) genetic cluster (B.C. and Yukon sequences only), highlighting five genetically distinct phylogenies that were in circulation during the first wave of the H5N1 HPAI outbreak in B.C./Yukon. Sequences in this timed tree are plotted based on specimen collection date. Trees are rooted by the KU201896 (H5) reference sequence.

Of the 144 H5 viruses detected, 113 generated high-quality sequence data, and all were confirmed as H5N1 viruses belonging to clade 2.3.4.4b. The highly basic HPAI motif in the HA cleavage site was also present in all H5N1 sequences that met established quality thresholds. To compare the viruses detected in B.C./Yukon with other sequenced cases in North America, we constructed an HA-specific phylogenetic tree consisting of local and publicly available H5N1 sequences from Canada and the United States. The first case of HPAI H5N1 in B.C. was detected on April 12, 2022, in a commercial poultry farm and HA-specific phylogenetic analysis indicated that this virus was closely related to HPAI viruses from Washington, Montana, Idaho, Wyoming, and Alaska (Figure 3(a)). Further examination of the B.C. data, in relation to the North American GISAID data, revealed that the B.C./Yukon sequences formed five distinct genetic clusters, each appearing to have different North American sequences as their closest relatives (Figure 3(b)). Presence of these distinct clusters suggests that multiple HPAI genotypes were co-circulating in B.C. and Yukon between April and September 2022.

**Figure 4:**
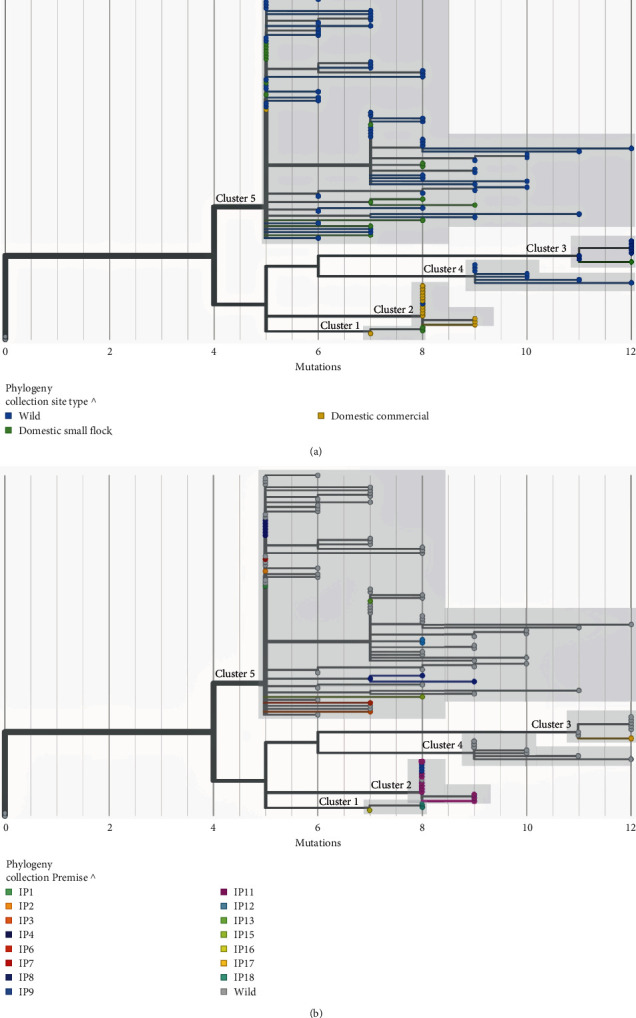
Membership within hemagglutinin (HA)-specific genetic clusters suggest different modes of transmission occurred during the British Columbia (B.C.)/Yukon outbreak. This is demonstrated by (a) different collection site type distribution (wild, domestic commercial, domestic-small flock) among genetic clusters and (b) presence of clusters of closely related viruses from different farms (infected premises). IP, infected premise. Gray tree tips represent wild birds. Trees are rooted by the earliest N. American H5N1 detection in the 2021/2022 outbreak, the A/chicken/NL/FAV-0033/2021 (H5) reference sequence.

Genomic surveillance of AIV in wild and domestic animals can enhance our understanding of sources and spread of HPAI across a landscape. To ascertain the probable routes of transmission among wildlife and domestic animals infected with HPAI, we examined the membership of the five genetically distinct clusters to determine whether infected animals from the same and/or different collection site types (Wild (avian or mammal), Domestic commercial, and Domestic small flock; Figure 4(a) and Figure S3) were genetically related. Genetic Cluster 1 contained only sequences from domestic birds, from one commercial farm and one small flock (Figure 4(b)). Samples from these two IPs were collected in similar space and time (Figures S3–S5). Cluster 2 was composed of sequences from two commercial poultry farms, and one wild bird detection. Notably, samples from the two commercial premises were collected from the same month and geographic area, however the wild bird was detected over a month later (Figures S3–S5). The genetic and epidemiological linkages between cases in Clusters 1 (*n* = 3) and 2 (*n* = 14) suggest that lateral transmission between the domestic IPs that shared the same genotype (i.e., Cluster 1 or 2) was plausible. In contrast, Cluster 4 (*n* = 7) contained only wildlife detections (6 wild birds, 1 wild mammal—red fox; *Vulpes vulpes*), while Clusters 3 (*n* = 8) and 5 (*n* = 81) represented viruses that had been detected largely in wildlife but also in some domestic birds. The largest genetic cluster, and most significant contributor to the B.C./Yukon outbreak within the study period was Cluster 5, which contained 18 domestic farms, 62 wild birds, and 1 wild mammal (striped skunk; *Mephitis mephitis*). Clusters 3 and 5 had mixed membership, indicating possible transmission of the virus between wild and domestic populations. However, available data did not allow for determination of the extent or directionality of these events.

#### 3.2.2. Genome-Wide Reassortant Characterization

Next, we examined whether the phylogenetic relationships identified in the HA-specific analysis were maintained across the remaining seven segments of the viral genome to evaluate whether HA-focused analyses could be used to infer genetic relatedness between sequences across the whole genome. Although the divergence between clusters varied depending on the segment, except for matrix (where sequences were more conserved, rendering Clusters 2 and 5 indistinguishable from each other), the membership of the five genetic clusters identified as distinct HA phylogeny (Figure 3(a)) were maintained across all other gene segments (Figure S6(a)–S6(h).

**Figure 5:**
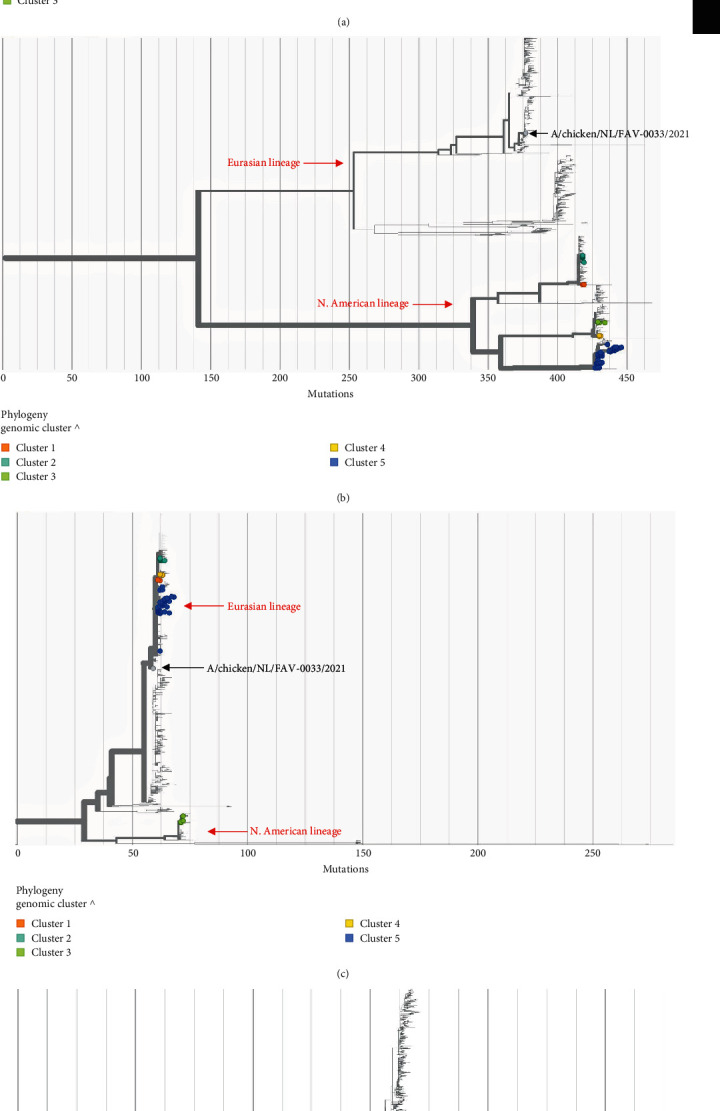
Contextualization of British Columbia (B.C.)/Yukon reassortants with global H5N1 sequences in GISAID between September 2021 and September 2022. B.C./Yukon genetic clusters relative to global GISAID sequences and the fully Eurasian A/chicken/NL/FAV-0033/2021 sequence detected at the start of the N. American outbreak highlight N. American reassortants in some or all of the clusters in NP (a), PB2 (b), NS (c), and PB1 (d) segments of the virus. Trees are rooted by the KU201896 (H5) reference sequence.

Examining all eight influenza A virus gene segments independently can shed light on the origins of the HPAI viruses introduced into B.C./Yukon over the course of the outbreak. Analysis of all gene segments for each of the five genetic clusters revealed two different genome constellation patterns (Table 2)—neither of which resembled the original Eurasian virus detected in Newfoundland in late 2021. Compared to the fully Eurasian (EA) Newfoundland virus, B.C./Yukon viruses, regardless of genetic cluster, were considerably divergent in both the NP and PB2 genome segments (Figures S6(d) and S6(h)) indicating that all B.C./Yukon sequences were NP and PB2 reassortants. Contextualization of our sequences with global GISAID NP and PB2 sequences from 2021–2022 revealed B.C./Yukon viruses clustered with North American (NAm) lineage LPAI viruses, suggesting B.C./Yukon reassortants were of NAm origin (Figures 5(a) and 5(b)). HPAI viruses belonging to Cluster 3 had two additional genome reassortments in NS and PB1 (Figures S6(e) and S6(g)). Constructing phylogeny with B.C./Yukon sequences and global GISAID sequences representing NS and PB1 segments of H5N1 viruses suggested that NS and PB1 segments belonging to Cluster 3 viruses were NAm in origin compared to viruses in Clusters 1, 2, 4, and 5, which had NS and PB1 segments that belonged to the EA lineage (Figures 5(c) and 5(d)).

### 3.3. Phylogeographic Analysis

Clusters 4 and 5 were the most common and were widely geographically dispersed (Figures S4 and S5). Cluster 5 was almost always the predominant cluster in every ecoprovince, regardless of the total number of overall cases (Figures S4 and S5). The exceptions were in May in the “Central Interior” (“CEI”), where Cluster 3 predominated in a group of American white pelicans (*P. erythrorhynchos*; *n* = 4), in May in the “Sub-boreal interior” (“SBI”) which had one case of Cluster 4 in a “Mammal,” in June in “CEI” where there was one case of Cluster 4 in a “Waterbird”; and in June in the “Southern Interior Mountains” (SIM) where there was one case of Cluster 2 in a “Raptor” (Figures S4 and S5). Cluster 1 was geographically isolated to “GED”, Cluster 2 only occurred in two ecoprovinces: “GED” and “SIM”. Cluster 3 was found in the Yukon, “CEI” and “GED”.

## 4. Discussion

For the “first wave” of the HPAI outbreak in western Canada (B.C. and the Yukon) between April 12 and September 11, 2022, our results demonstrate an unprecedented level of taxonomic diversity—the number of bird species and functional groups—affected by morbidity and mortality attributable to HPAI H5N1 2.3.4.4b. This differs from previous outbreaks in Canada; however, the impacts on wildlife associated with this outbreak are consistent with findings in Europe between 2016 and 2022 [37], including Germany [38], France [39], and the U.S.A. in 2022 [4, 22].

The largest proportion of wild bird cases in this study occurred in Canada geese (“Waterfowl”). It is possible that some Canada geese were misidentified and were cackling geese (*Branta hutchinsii*), a smaller, but remarkably similar bird in appearance. Canada geese are highly gregarious, often residing in large groups with close contacts, which makes large localized, and visible, outbreaks possible. However, few studies have examined why Canada geese are particularly susceptible to H5N1. Harris et al. [40] and Giacinti et al. [20] determined that Canada geese are unlikely reservoirs for low pathogenicity AIVs due to brief shedding patterns or low rates of positivity, but Pasick et al. [41] determined that, experimentally, Canada geese are susceptible to HPAI infection, and Neufeld et al. [42] found that this was especially true for juvenile animals. During an outbreak of H5N1 in Germany in 2006, some Canada geese were infected at lower rates than other waterfowl species [43], but this may simply reflect lower densities of Canada geese in this region. During an HPAI outbreak in Europe between September 10 and December 2, 2022, only 60/613 (9.8%) of wild bird detections were in Canada geese [37]. However, Giacinti et al. [20] demonstrated that Canada geese were also the most common species to test positive for HPAI nationally during this time period. The high numbers of HPAI detections that we observed in Canada geese may have been due to a combination of factors related to their age class, behavioral dynamics, environmental contamination, viral genotype susceptibility, or our opportunistic sampling approach with sample prioritization. It is possible that specific genotypes of the virus may have caused higher mortality (discussed later) and note that all Canada geese in this study belonged to a single phylogenetic cluster (Cluster 5). While Canada geese are unlikely potential reservoirs for HPAI in North America, they may potentially serve as an early and persistent warning system.

The next most common wild species cases in our dataset were two species of “Raptor”: bald eagles and great horned owls. The same was true for the outbreak nationally [20]. The majority of “Raptor” cases occurred in the first half of the outbreak described, and sometimes in comparable quantities as “Waterfowl” cases (Figure S2). However, this may have been influenced by sampling (carcass collection) strategy, which sometimes prioritized new geographic areas. During the 2014–2015 outbreak in the U.S.A., raptor species were found to be highly susceptible to infection with clade 2.3.4.4b viruses, with six different species affected [44]. Further, 136 bald eagles experienced HPAI-related mortality in the Southeastern U.S.A. between January and June 2022 [5]. Raptors most likely become infected by consuming prey that were sick or had died from HPAI. Bald eagles, in particular, which frequent peri-aquatic habitats and tend to be opportunistic foragers that readily select moribund or deceased birds as an easy food source [45], but great horned owls have also been documented to display opportunistic scavenging behaviors [46].

The degree to which data from passive surveillance is representative of the true epidemiology and disease ecology of HPAI H5N1 in wildlife is unknown. However, this sampling bias toward large geese and bald eagles may suggest that our dataset is missing smaller bird species that are either more difficult to spot, less likely to be found in urban areas, or are more quickly removed from the landscape by predators. This is supported by the surge of raptor cases observed prior to detections in waterfowl, as raptors were likely to consume infected birds that we were not able to detect via passive surveillance strategies prior to their consumption by predators. Furthermore, passive surveillance strategies will not detect birds that are infected but not sick and dying, and likely mobile, contributing to further widespread geographic transmission of the virus [9, 47]. Indeed, it is of note that our dataset contains very few dabbling ducks such as mallards (*Anas platyrhynchos*), green-winged teals (*Anas carolinensis*), northern pintails (*Anas acuta*), American wigeon (*Mareca americana*), as well as the other dabbling ducks and most Charadriiformes, which have often been described as carriers of low pathogenicity AIVs [12] and likely also effective spreaders of HPAI across the global landscape [48]. In the latter summer months, decreased detections in these species is also likely due to their migratory patterns which send them to their remote northern breeding grounds outside of our study region. It is of note that the biases associated with passive surveillance also make it difficult to compare results between studies and surveillance programs. For example, people who report dead birds as well as collection agencies may prioritize certain places and species as an outbreak goes on, leading to certain species being overrepresented.

A consistent geographic pattern is absent when we examine the proportions of functional groups detected within this region and between ecoprovinces. These results differ from some other jurisdictions, such as southwestern France in 2016–2017, which experienced strong geo-clustering of the HPAI genotypes identified [49]. The complexity in the observed case distribution may be related to the possibility that the virus is maintained in multispecies metapopulations [50]—as in subpopulations of animals connected by movement which may be impacted by or consist of migratory species, resident birds, and environmental contamination [51].

It is of note that the majority of the samples analyzed in wave one of the B.C./Yukon outbreak came from more densely populated areas (by humans) of the province/territory. Passive surveillance relies heavily on human detections of cases—i.e., people observing sick or dead birds, and then the logistic capabilities of involved parties to retrieve carcasses. Thus, while we saw fewer detections across northern B.C. and the Yukon, it is highly likely that this is due to under sampling, rather than a true lack of cases across these geographic regions. That being said, strong inter-agency partnerships with regional conservation officers meant that larger scale mortality events were likely detected in the majority of cases, even in remote areas. Overall, the complex disease ecology of HPAI, in combination with under sampling across a very large geographic area, make it difficult to determine how exactly wild bird detections can be translated into local and regional domestic poultry, and wild bird infection risk.

Regarding temporal dynamics, we saw a peak in cases in May, and another, albeit smaller, spike in cases in wild birds (predominantly “Waterfowl”) in August. Cases in domestic birds and mammals were only detected in the first half of this outbreak, and cases in mammals across B.C. and the Yukon were only detected in May. This likely corresponds to the highest densities of migratory reservoir species during their spring migration north to places such as Alaska via the Pacific flyway [10].

Phylogenetic analysis of the HA segment confirmed that all positive detections within the study period belonged to HPAI clade 2.3.4.4b, and were closely related to earlier detections of HPAI in the U.S.A. Notably, B.C. sequences were considered more closely related to U.S.A. sequences from the northwestern states (e.g., Washington, Montana, Idaho, etc.) than to other Canadian provinces or U.S. states suggesting the virus was introduced by migratory birds entering B.C. via the Pacific Flyway. This interpretation is supported by: (1) evidence that the root-to-tip distances of H5 sequences from B.C. are more evolved than H5 sequences from the northwestern U.S. states (based on available public data); and (2) the observation that B.C. H5 sequences form monophyletic clades with H5 sequences from the northwestern U.S. states, relative to H5 sequences identified in the other U.S. states. Further examination of the diversity of the B.C./Yukon sequences, and their relatedness to U.S.A. detections highlighted the presence of five genetically distinct clusters of HPAI viruses, indicating that multiple incursion (or introduction) events into B.C./Yukon likely occurred during this outbreak.

Analyzing all eight segments of the B.C./Yukon viruses relative to global H5N1 GISAID sequences from 2021–2022 revealed the presence of two genome constellations, both of which were genetically distinct from the fully Eurasian A/chicken/NL/FAV-0033/2021 virus detected in Eastern Canada at the start of the North American outbreak [19]. Relative to A/chicken/NL/FAV-0033/2021, B.C./Yukon viruses had acquired 2–4 gene segments from NAm lineage LPAI viruses detected in the U.S.A., further supporting the hypothesis that B.C./Yukon viruses had undergone reassortment with *N*. American lineages in the U.S.A. prior to their arrival in Western Canada. Genome constellations provide one layer of resolution when characterizing the diversity of viruses circulating during an AIV outbreak. In this study, we show that although Clusters 1, 2, 4, and 5 were represented by the same genome constellation, they were considered genetically distinct phylogeny across all segments (except M, which is highly conserved), suggesting that we cannot assume all samples with the same genome constellation belong to the same or similar genotypes. The five genetic clusters differed based on membership (by functional group and collection site type), size (*N*), and geographic distribution suggesting that multiple factors are shaping the phylodynamics of this outbreak. These findings illustrate how in-depth genetic characterization of HPAI can enhance our understanding of the diversity and origins of circulating viruses, transmission dynamics, and risks to animal and human health, all of which are key parameters for effective HPAI surveillance.

Despite identifying five distinct viral genotypes belonging to clade 2.3.4.4b in our dataset, Cluster 5 was by far the most dominant HPAI subclade detected during the “first wave” of the B.C./Yukon outbreak. Several factors may have led to the predominance of Cluster 5 including viral fitness, host susceptibility, environmental conditions, or founder effects. We also must consider that a combination of these factors may have contributed to a biased sampling approach; limited by our passive surveillance strategy, a more virulent virus would lead to more deaths, which would have been detected more readily than one that caused less mortality. Identifying these factors can inform disease control strategies for both wildlife and poultry, and help mitigate the impact of future outbreaks in affected populations, thus work to further characterize the genetic and pathological effects of these divergent viruses is necessary.

Historically, the Sanger sequencing method was used for genetic characterization of influenza viruses, and much of this work focused primarily on the highly antigenic HA segment [52]. Since, WGS has replaced Sanger, phylogenetic analysis of the whole genome is possible by: (1) analyzing concatenated segments or (2) analyzing each segment individually. However, longer segments are difficult to sequence entirely, meaning that large parts of the genome would have to be excluded or these samples removed entirely from the analysis. For that reason, we chose to analyze each segment individually, and only segments that individually passed QC were included in the analysis. Due to these challenges, we investigated whether the genetic diversity within HA alone could be used as a proxy to define relationships between HPAI cases. As discussed above, we found that membership for each genomic cluster identified in the HA phylogenetic tree was maintained across all 8 segments, with the exception of Clusters 2 and 5 for M gene, which were indistinguishable, suggesting that HA could be used as a “first pass” to identify the majority of the genetic relationships among circulating viruses. While a lot can be gleaned from HA-specific analysis, analyzing the whole genome is also important and should not be overlooked, as the internal gene segments (i.e., M, NP, NS, PB2, PB1, PA) are more prone to reassortment and can acquire key mutations that confer mammalian adaptation [53].

Generally, we observed higher genomic cluster and functional group diversity (i.e., more clusters and more functional groups represented) in locations with more cases (Figures S1−S5). The exception was an American white pelican outbreak in the CEI ecoprovince, where one case in April (unsequenced), four cases in May (all Cluster 3), and one case in June (Cluster 4) occurred (Figure S5). Comparing a larger geographic area or examining a more evenly distributed dataset might reveal more pronounced spatiotemporal patterns in the circulating HPAI viruses. For instance, Alkie et al. [54] and Giacinti et al. [20] found distinct reassortment pattern clustering in 2022 Canadian mammalian samples which correlated geographically with specific avian migratory flyways across North America (e.g., Central, Mississippi, and Atlantic). Giacinti et al. [20] also found a flyway effect in bird samples. Alternatively, a less distinctive geospatial trend in our data could simply reflect the diversity in co-circulating AIVs during this outbreak within a specific flyway (Pacific) or reflect limitations of the passive surveillance strategy.

With regard to HPAI in domestic birds, in the first wave of the B.C./Yukon outbreak we observed a greater number of small domestic flocks impacted than commercial poultry flocks. While the risk of transmission between small flocks and commercial flocks remains to be established in this outbreak, a similar trend was identified in an outbreak of H5N8 clade 2.3.4.4 in France in 2016–2017 [55]. Small flocks may be at increased risk for HPAI compared to commercial farms because of decreased knowledge about or implementation of biosecurity measures [56], and potentially increased contact with wild birds due to free-range practices. Our data do not demonstrate that small poultry flocks pose any increased risk to commercial flocks. In our dataset there was only one instance where commercial and small flock premises were infected with closely related viruses (Cluster 1); examining the root-to-tip distances of the H5 segments from both premises indicated that the small flock viruses were more evolved than the virus detected on the commercial farm, suggesting that lateral transmission from small flock to commercial farm was unlikely, however epidemiological traceback analysis would be required for detailed interpretation of these genetic data.

Indeed, there were relatively few farms infected with closely related HPAI viruses, the exception being the farms in Clusters 1 and 2. However, the limitations of the surveillance strategy in wild birds mean that even when farms are infected with closely related viruses, we cannot rule out the possibility that wild bird carriers were responsible for infecting farms with similar viruses, as opposed to farm-to-farm spread. A limitation of the domestic bird surveillance strategy is that HPAI-positive samples are only collected at the onset of an outbreak, not throughout depopulation. Therefore, we are unable to capture the full extent of viral evolution and viral diversity during a premise outbreak. An enhanced surveillance strategy, achieved by sampling a larger number of birds from the initial detection period through depopulation could improve resolution of transmission chains between IPs and proximal wildlife populations.

## 5. Conclusion

Our study reveals the diverse taxonomy and geographic scope of wild birds and mammals affected by an H5N1 outbreak in B.C. and the Yukon. We observed a temporal trend in functional groups and an extended detection period into summer and early fall. In most cases, the genetic diversity of HPAI viruses detected by WGS analysis was not strongly associated with geography, but instead found that some genotypes were more widespread (across space and time) than others, although the reason for this is currently unknown. We detected two different genome constellations that represented reassortants in 2–4 segments of the genome, which were North American in origin (compared to the others which were Eurasian in origin). These viruses were further subdivided into five distinct genotypes, which were consistently different across all eight gene segments, and displayed their own phylodynamic characteristics. Additionally, examining the relationships between these genotypes and closely related viruses from the northwestern U.S. states suggest multiple HPAI viruses were likely introduced into western Canada by migratory birds via the Pacific Flyway. These findings underscore the ability of WGS to add another layer of resolution to our understanding of the ecology of HPAI outbreaks.

We recommend the continuation of active live bird and/or environmental surveillance in wild and domestic animals in order to address some of the shortfalls discussed above, particularly the uncertainty regarding the degree to which passive surveillance data reflects the true ecology and epidemiology of HPAI in wild birds [37]. Regardless, integrating epidemiological, geographic, and phylogenetic data from wild and domestic animals are crucial for continued assessment of HPAI risk and intervention planning in poultry production. It can also help inform risks to wildlife and public health through consistent monitoring of HPAI mutations that enhance viral fitness, increase virulence in certain species, or cause more severe disease in wildlife and humans. Finally, this work supports our understanding of HPAI evolution, contributes to enhanced global surveillance networks, and aids in the development of effective control strategies designed to protect both animal and human health.

## Figures and Tables

**Table 1:** Distribution of wild species (birds and mammals) that tested positive for highly pathogenic avian influenza (HPAI; H5N1 clade 2.3.4.4b) in British Columbia (B.C.) and the Yukon during the “first wave” of the outbreak in this region, between April 12 and September 11, 2022.

Species	Functional group	April	May	June	July	August	September	Total *N*
Avian spp.								
Canada goose (*B. canadensis*)	Waterfowl	2	11	15	4	13	2	47
Bald eagle (*H. leucocephalus*)	Raptor	10	4	9	1	1	0	25
Great horned owl (*B. virginianus*)	Raptor	5	6	1	0	0	0	12
American white pelican (*P. erythrorhynchos*)	Waterbird	1	4	1	0	0	0	6
Great blue heron (*Ardea herodias*)	Waterbird	1	2	1	0	1	0	5
Snow goose (*Anser caerulescens*)	Waterfowl	3	2	0	0	0	0	5
Northwestern crow (*Corvus caurinus*)	Landbird	0	3	0	0	0	0	3
Wood duck (*Aix sponsa*)	Waterfowl	0	2	0	0	1	0	3
Common raven (*Corvus corax*)	Landbird	0	2	0	0	0	0	2
Cooper's hawk (*Accipiter cooperii*)	Raptor	1	0	1	0	0	0	2
Peregrine falcon (*Falco peregrinus*)	Raptor	0	2	0	0	0	0	2
Mallard (*A. platyrhynchos*)	Waterfowl	0	2	0	0	0	0	2
Northern shoveler (*Spatula clypeata*)	Waterfowl	0	0	0	0	2	0	2
Pine siskin (*Spinus pinus*)	Landbird	1	0	0	0	0	0	1
Common barn owl (*Tyto alba*)	Raptor	0	1	0	0	0	0	1
Red-tailed hawk (*Buteo jamaicensis*)	Raptor	1	0	0	0	0	0	1
Turkey vulture (*Cathartes aura*)	Raptor	0	0	1	0	0	0	1
Glaucous-winged gull (*Larus glaucescens*)	Waterbird	0	1	0	0	0	0	1
Red-breasted merganser (*Mergus serrator*)	Waterbird	0	0	0	0	1	0	1
Red-necked grebe (*Podiceps grisegena*)	Waterbird	1	0	0	0	0	0	1
Trumpeter swan (*C. buccinator*)	Waterfowl	0	1	0	0	0	0	1
Mammalian spp.								
Red fox (*V. vulpes*)	Mammal	0	3	0	0	0	0	3
Striped skunk (*M. mephitis*)	Mammal	0	1	0	0	0	0	1

**Table 2:** Reassortant classification and genome constellations corresponding with the five genetic clusters identified among the H5N1 viruses in the British Columbia (B.C.)/Yukon outbreak.

Influenza A segment									
Genetic cluster	HA	NA	M	NP	NS	PA	PB1	PB2	Genome constellation
Cluster 1	EA	EA	EA	NAm	EA	EA	EA	NAm	A
Cluster 2	EA	EA	EA	NAm	EA	EA	EA	NAm	A
Cluster 3	EA	EA	EA	NAm	NAm	EA	NAm	NAm	B
Cluster 4	EA	EA	EA	NAm	EA	EA	EA	NAm	A
Cluster 5	EA	EA	EA	NAm	EA	EA	EA	NAm	A

This classification was supported by phylogenetic analyses incorporating global GISAID sequences into the phylogeny with B.C./Yukon sequences for each virus segment. EA, Eurasian lineage; NAm, North American lineage.

## Data Availability

The sequence data that support the findings of this study are publicly available on GISAID (https://www.gisaid.org/).

## Supplementary Materials

Supplementary Materials Additional supporting information can be found online in the supporting information section at the end of this article. Figure S1: map with proportional pie charts, per ecoprovince, per functional group, total outbreak. Figure S2: maps with proportional pie charts, per ecoprovince, per functional group, per month. Figure S3: number of whole genome sequences per functional group, per month. Figure S4: map with proportional pie charts, per whole genome cluster, per ecoprovince, total outbreak. Figure S5: map with proportional pie charts, per whole genome cluster, per ecoprovince, per month. Figure S6: HA-specific genetic clusters are maintained across all eight segments (a–h), but there is evidence of reassortment.
