# Supplementary material for: Descriptive Epidemiology and Phylodynamics of the “First Wave” of an Outbreak of Highly Pathogenic Avian Influenza (H5N1 Clade 2.3.4.4b) in British Columbia and the Yukon, Canada, April to September 2022
Source: Transbound Emerg Dis. 2024 Feb 29;2024:2327939. doi: 10.1155/2024/2327939 (PMC12017231; doi:10.1155/2024/2327939)
Supplement: Supplementary Materials — Additional supporting information can be found online in the supporting information section at the end of this article. Figure S1: map with proportional pie charts, per ecoprovince, per functional group, total outbreak. Figure S2: maps with proportional pie charts, per ecoprovince, per functional group, per month. Figure S3: number of whole genome sequences per functional group, per month. Figure S4: map with proportional pie charts, per whole genome cluster, per ecoprovince, total outbreak. Figure S5: map with proportional pie charts, per whole genome cluster, per ecoprovince, per month. Figure S6: HA-specific genetic clusters are maintained across all eight segments (a–h), but there is evidence of reassortment. [file 2327939.f1.docx]

**SUPPLEMENTARY MATERIALS**


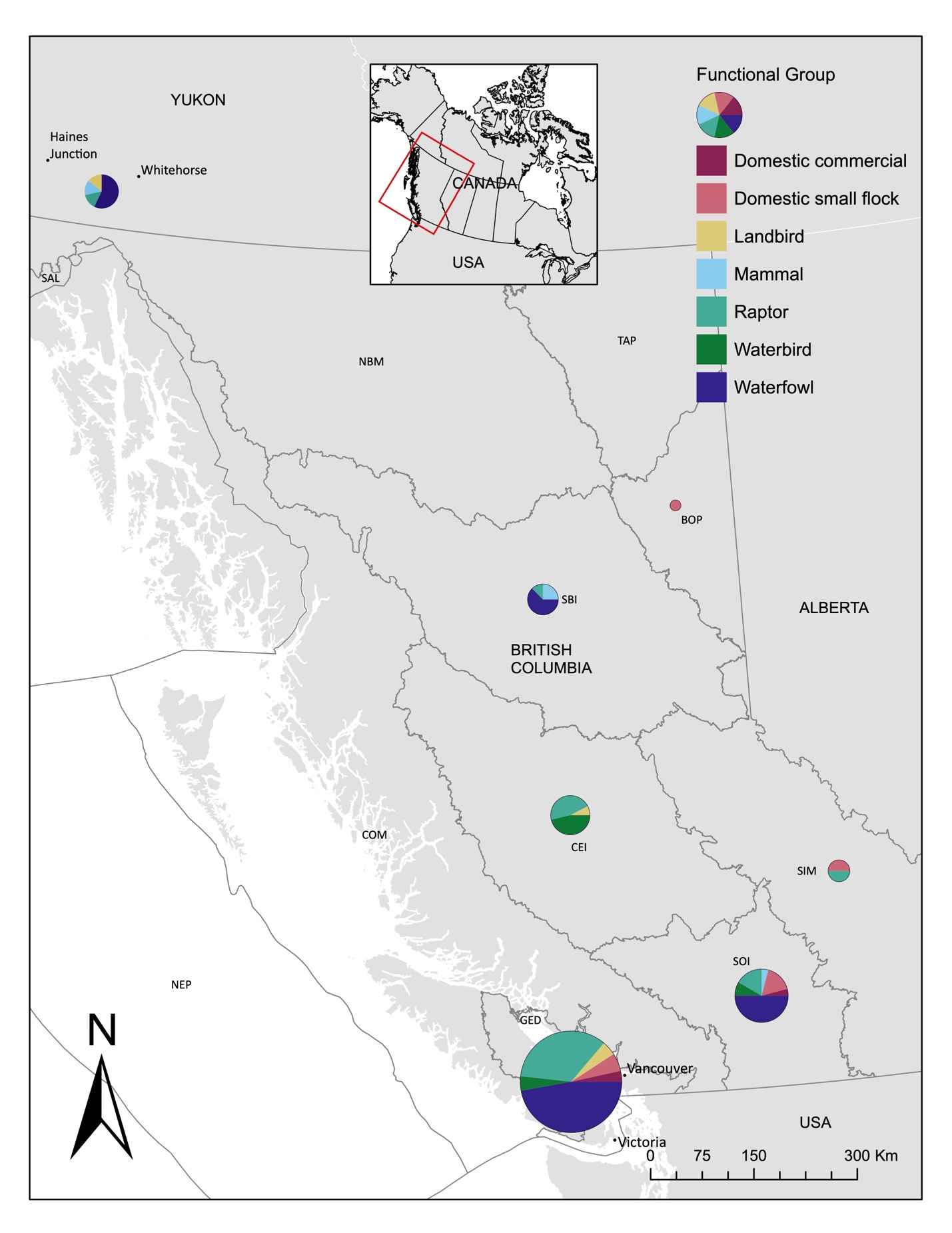


**FIGURE S1** Map with proportional pie charts representing proportions of cases of highly pathogenic avian influenza (HPAI; H5N1 clade 2.3.4.4b) in British Columbia (B.C.) and the Yukon, Canada, during the ‘first wave’ of the outbreak in this region, between April 12 and September 11, 2022. Data is categorized per ‘Functional Group’ (‘Domestic commercial’, ‘Domestic small flock’, ‘Landbird’, ‘Mammal’, ‘Raptor’, ‘Waterbird’, or ‘Waterfowl’) and per ecoprovince (SAL: Southern Alaska Mountains; NBM: Northern Boreal Mountains; TAP: Taiga Plains; NEP: Northeast Pacific; COM: Coast and Mountains; SBI: Sub-boreal Interior; BOP: Boreal Plains; CEI: Central Interior; GED: Georgia Depression; SOI: Southern Interior; SIM: Southern Interior Mountains).

**
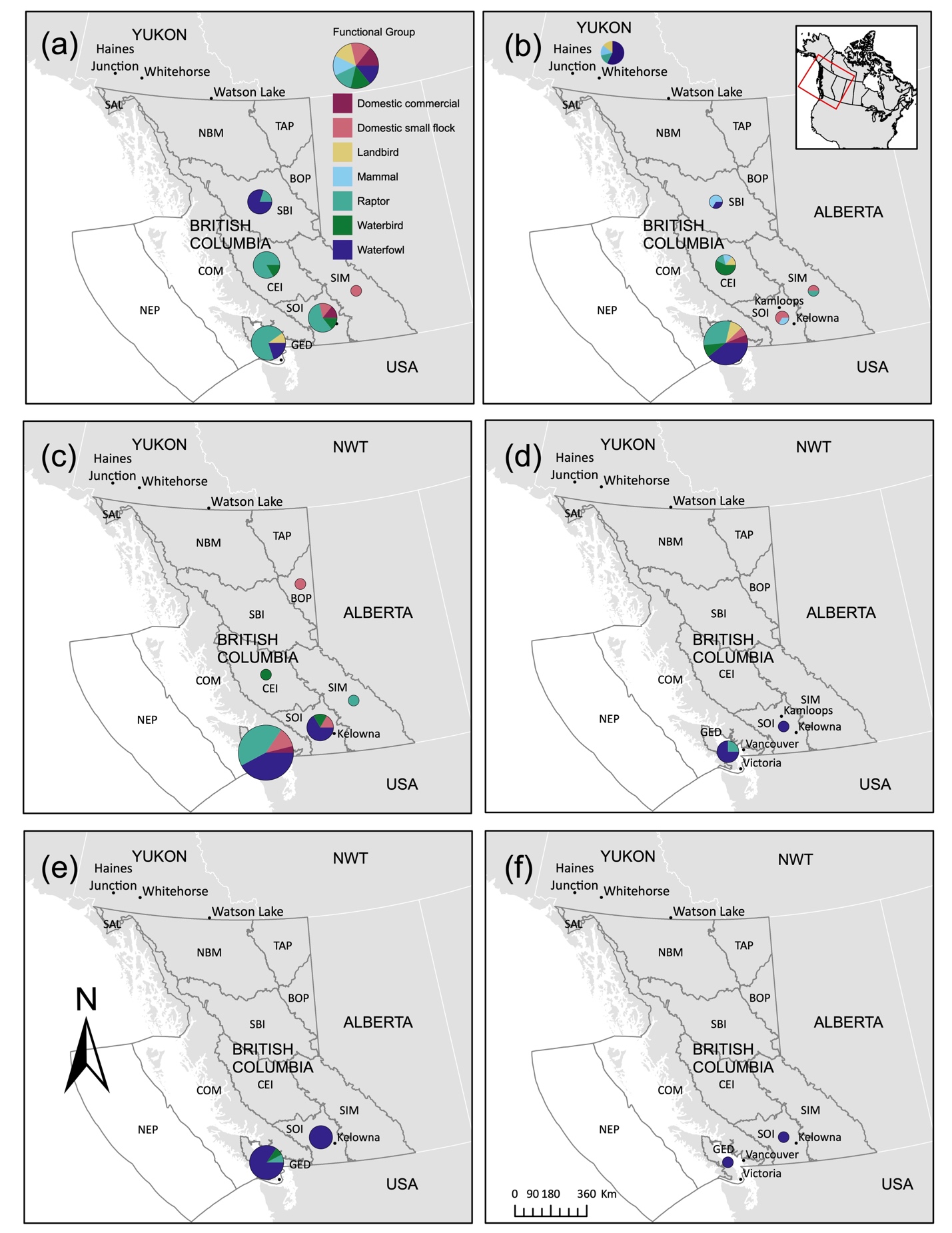
**

**FIGURE S2** Maps with proportional pie charts displaying proportions of cases of highly pathogenic avian influenza (HPAI; H5N1 clade 2.3.4.4b) in British Columbia (B.C.) and the Yukon, Canada, during the ‘first wave’ of the outbreak in this region, between April 12 and September 11, 2022, per month. Data is characterized by ‘Functional Group’ (‘Domestic commercial’, ‘Domestic small flock’, ‘Landbird’, ‘Mammal’, ‘Raptor’, ‘Waterbird’, or ‘Waterfowl’) per month (April(a), May (b), June (c), July (d), August (e), and September (f)), and per ecoprovince (SAL: Southern Alaska Mountains; NBM: Northern Boreal Mountains; TAP: Taiga Plains; NEP: Northeast Pacific; COM: Coast and Mountains; SBI: Sub-boreal Interior; BOP: Boreal Plains; CEI: Central Interior; GED: Georgia Depression; SOI: Southern Interior; SIM: Southern Interior Mountains).


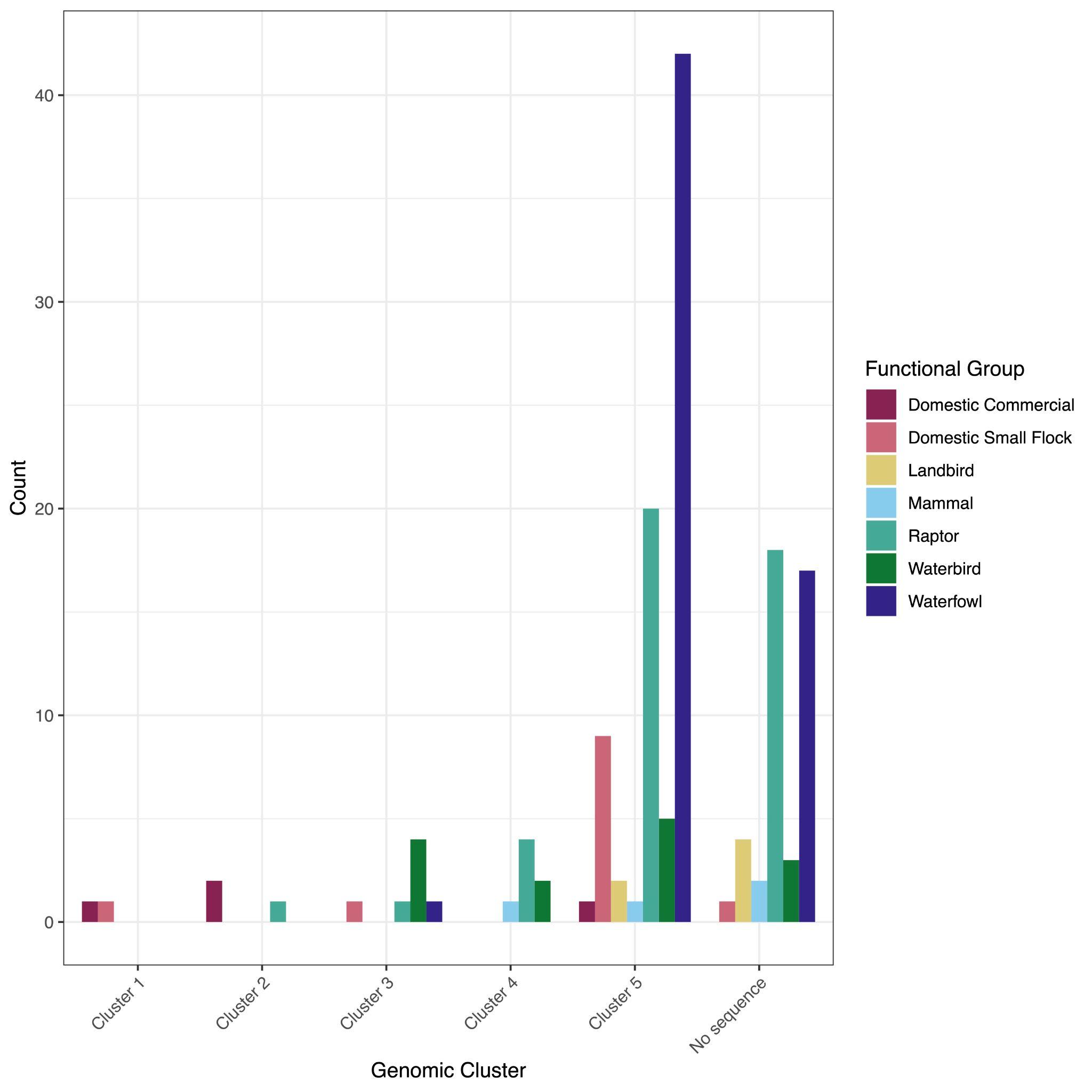


**FIGURE S3** Number of whole genome sequences of highly pathogenic avian influenza (HPAI; H5N1 clade 2.3.4.4b) detected in British Columbia (B.C.) and the Yukon, Canada, during the ‘first wave’ of the outbreak in this region, between April 12 and September 11, 2022 per cluster (1-5) and per ‘Functional Group’ (‘Domestic commercial’, ‘Domestic small flock’, ‘Landbird’, ‘Mammal’, ‘Raptor’, ‘Waterbird’, or ‘Waterfowl’).


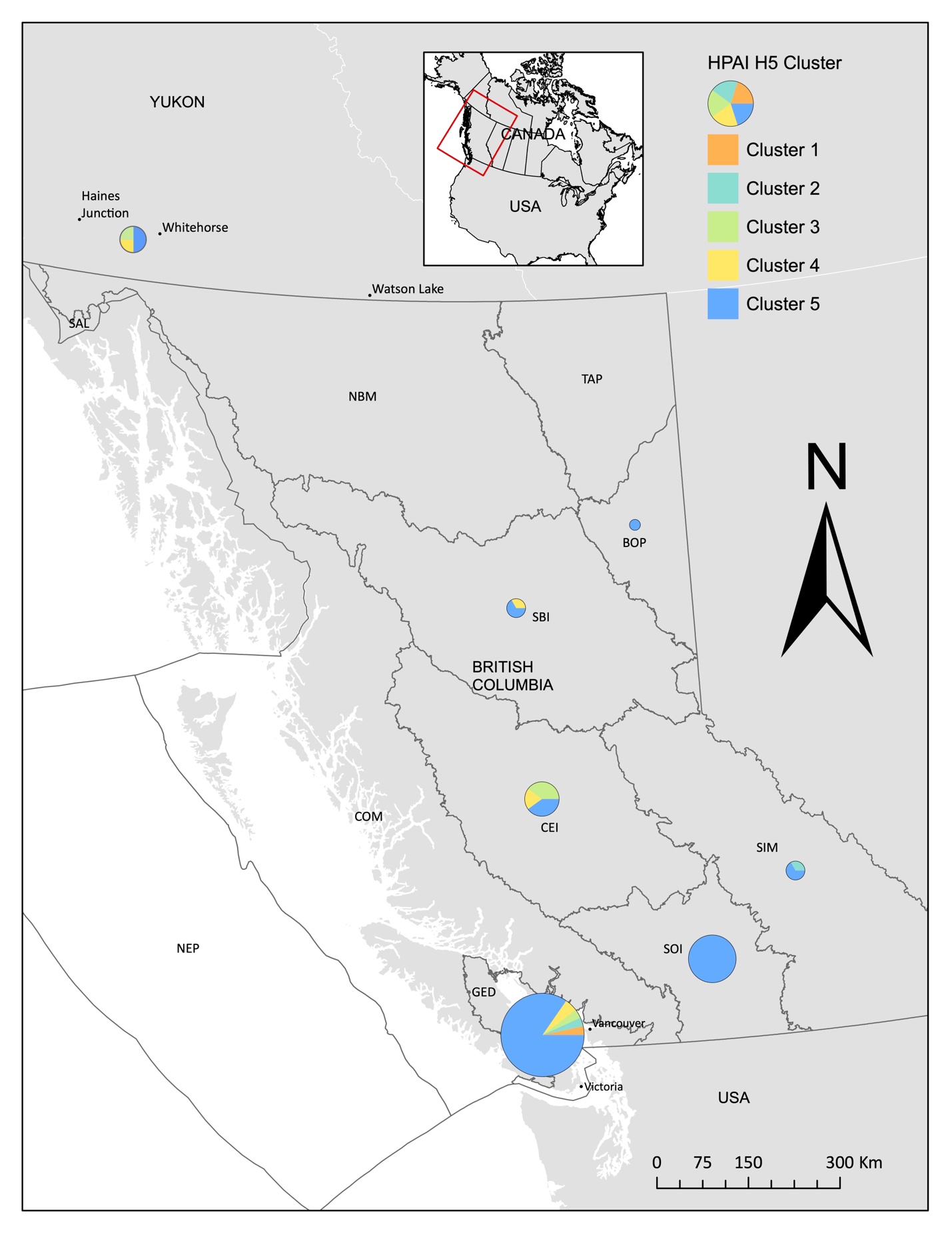


**FIGURE S4** Map with proportional pie charts representing proportions of samples successfully whole genome sequenced for highly pathogenic avian influenza (HPAI; H5N1 clade 2.3.4.4b) in British Columbia (B.C.) and the Yukon, Canada, during the ‘first wave’ of the outbreak in this region, between April 12 and September 11, 2022. Proportions are classified by genomic cluster (1-5) and per ecoprovince (SAL: Southern Alaska Mountains; NBM: Northern Boreal Mountains; TAP: Taiga Plains; NEP: Northeast Pacific; COM: Coast and Mountains; SBI: Sub-boreal Interior; BOP: Boreal Plains; CEI: Central Interior; GED: Georgia Depression; SOI: Southern Interior; SIM: Southern Interior Mountains).


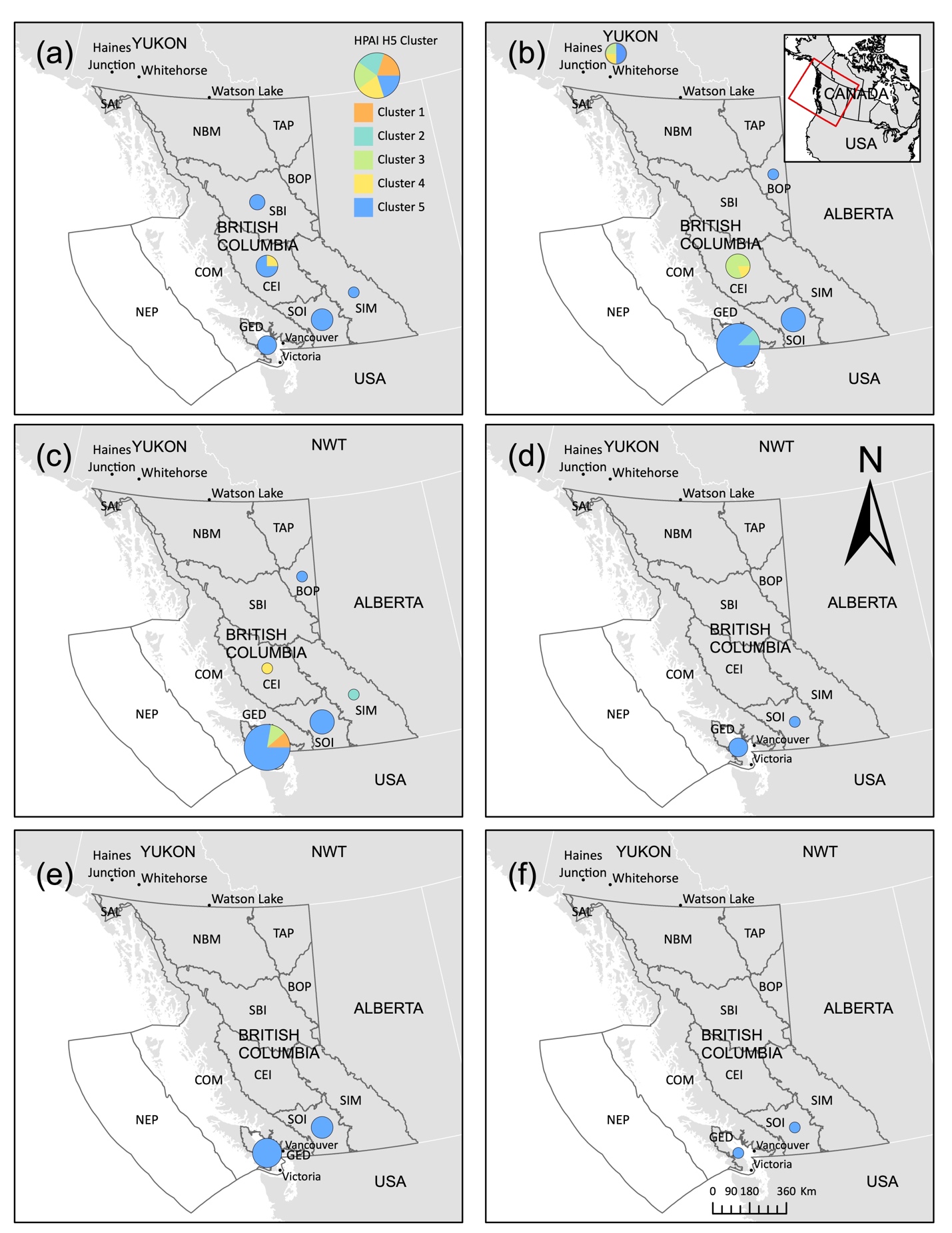


**FIGURE S5** Map with proportional pie charts representing proportions of samples successfully whole genome sequenced for highly pathogenic avian influenza (HPAI; H5N1 clade 2.3.4.4b) in British Columbia (B.C.) and the Yukon, Canada, during the ‘first wave’ of the outbreak in this region, between April 12 and September 11, 2022, per month. Proportions are classified by genomic cluster (1-5), per month (April(a), May (b), June (c), July (d), August (e), and September (f)), and per ecoprovince (SAL: Southern Alaska Mountains; NBM: Northern Boreal Mountains; TAP: Taiga Plains; NEP: Northeast Pacific; COM: Coast and Mountains; SBI: Sub-boreal Interior; BOP: Boreal Plains; CEI: Central Interior; GED: Georgia Depression; SOI: Southern Interior; SIM: Southern Interior Mountains).

**FIGURE S6** HA-specific genetic clusters are maintained across all 8 segments (A-H) of the H5N1 viruses in the British Columbia (B.C.)/Yukon outbreak, but there is evidence of reassortment. Relative to the fully Eurasian H5N1 root (A/chicken/NL/FAV-0033/2021), detections in B.C./Yukon display two unique genome constellations whereby (A) HA (B) NA, (C) M and (F) PB belong to the Eurasian lineage, (D) NP and (H) PB2 belong to the N. American lineage, and (E) NS and (G) PB1 belong to the Eurasian lineage, with the exception of samples in Cluster 3, which belong to the N. American lineage. Trees are rooted by the earliest N. American H5N1 detection in the 2021/2022 outbreak, the A/chicken/NL/FAV-0033/2021 (H5) reference sequence.
